# Telemedicine for Personalized Nutritional Intervention of Rare Diseases: A Narrative Review on Approaches, Impact, and Future Perspectives

**DOI:** 10.3390/nu17030455

**Published:** 2025-01-26

**Authors:** Francesca Eletti, Veronica Maria Tagi, Ilenia Pia Greco, Eliana Stucchi, Giulia Fiore, Eleonora Bonaventura, Fabio Bruschi, Davide Tonduti, Elvira Verduci, Gianvincenzo Zuccotti

**Affiliations:** 1 Department of Pediatrics, Vittore Buzzi Children’s Hospital, University of Milan, 20154 Milan, Italy; francesca.eletti@unimi.it (F.E.); veronica.tagi@unimi.it (V.M.T.); ilenia.greco@unimi.it (I.P.G.); eliana.stucchi@unimi.it (E.S.); giulia.fiore@unimi.it (G.F.); gianvincenzo.zuccotti@unimi.it (G.Z.); 2 Department of Biomedical and Clinical Science, University of Milan, 20157 Milan, Italydavide.tonduti@asst-fbf-sacco.it (D.T.); 3 Child Neurology Unit, Buzzi Children’s Hospital, 20154 Milano, Italy; eleonora.bonaventura@asst-fbf-sacco.it; 4 C.O.A.L.A. (Center for Diagnosis and Treatment of Leukodystrophies), Unit of Pediatric Neurology, V. Buzzi Children’s Hospital, 20154 Milan, Italy; 5 Department of Health Sciences, University of Milan, 20146 Milan, Italy; 6 Metabolic Diseases Unit, Department of Pediatrics, Vittore Buzzi Children’s Hospital, University of Milan, 20154 Milan, Italy

**Keywords:** telehealth, telemedicine, neurological impairment, nutrition, rare diseases, inherited metabolic diseases, artificial intelligence

## Abstract

**Background:** Telemedicine represents a growing opportunity to improve access to personalized care for patients with rare diseases, addressing the challenges of specialized healthcare that is often limited by geographical barriers. The aim of this narrative review is to explore how telemedicine can facilitate tailored nutritional interventions for rare diseases, focusing on inherited metabolic diseases, rare neurological disorders, such as leukodystrophies, and neuromuscular disorders, including spinal muscular atrophies. **Methods:** This narrative review is based on a systematic search of the published literature over the past 20 years, and includes systematic reviews, meta-analysis, retrospective studies, and original articles. References were selected through searches in databases such as PubMed and Scopus, applying predefined inclusion and exclusion criteria. Among the inclusion criteria, studies focusing on pediatric patients aged 0 to 18 years, diagnosed with rare neurological diseases or inherited metabolic disorders, and using telemedicine in addition to in-person visits at their reference center were considered. Among the exclusion criteria, studies involving patients with other pathologies or comorbidities and those involving patients older than 18 years were excluded. **Results:** A total of 66 documents were analyzed to examine the challenges and specific needs of patients with rare diseases, highlighting the advantages and limitations of telemedicine compared to traditional care. The use of telemedicine has revolutionized the medical approach, facilitating integrated care by multidisciplinary teams. **Conclusions:** Telemedicine still faces several technical, organizational, and security challenges, as well as disparities in access across different geographical areas. Emerging technologies such as artificial intelligence could positively transform the monitoring and management of patients with rare diseases. Telemedicine has great potential ahead of it in the development of increasingly personalized and effective care, in fact, emerging technologies are important to provide remote care, especially for patients with rare diseases.

## 1. Introduction

According to the World Health Organization (WHO), telemedicine is defined as “the delivery of healthcare services, where distance is a critical factor, by all healthcare professionals using information and communication technologies for the exchange of valid information for diagnosis, treatment and prevention of disease and injuries, research and evaluation, and for the continuing education of healthcare providers, all in the interests of advancing the health of individuals and their communities” [1]. This approach is particularly valuable for patients with rare diseases (RDs), especially when they reside far from specialized centers and face significant obstacles to timely and continuous care [2]. Accessing healthcare represents a real challenge for several children affected by rare diseases and their caregivers. Rare diseases are increasingly recognized as a global public health priority [3], highlighting that specialized centers are typically concentrated in urban areas, limiting care options for patients residing in rural zones. Many rare diseases also require multidisciplinary care, which involves collaboration among specialists such as neurologists, geneticists, nutritionists, and physiotherapists. Delays in diagnosis can be prolonged, and they lead to missed opportunities for early intervention, negatively impacting patient outcomes [4,5].

Some rare diseases, such as inborn errors of metabolism (IEM), require dietary therapies involving strict nutritional management and continuous monitoring. Conditions like urea cycle disorders (UCDs), phenylketonuria, aminoacidopathies, fatty acid oxidation deficiencies, organic acidemias, and carbohydrate metabolism disorders depend on specific dietary restrictions and supplementation with amino acids and other essential nutrients. Nutrition professionals play a critical role in ensuring adherence to these complex dietary regimens [6,7]. Managing such regimens is particularly burdensome for families, especially when patients are young and require ongoing assistance, including home-based blood testing and frequent consultations with specialists [7,8]. Emotional burden is another challenge faced by families of children with rare diseases, with isolation, anxiety, and depression exacerbated by stigma and delays [9]. Telemedicine has proven particularly beneficial during these challenges by supporting interdisciplinary care, enabling remote monitoring, and addressing barriers such as distance and limited access to medical consultations [2,10]. The COVID-19 pandemic has represented a considerable challenge in providing rehabilitation services for medically fragile patients with rare neuromuscular diseases such as Duchenne muscular dystrophy (DMD) and spinal muscular atrophy (SMA) [11]. The COVID-19 pandemic highlighted the importance of telemedicine, as many healthcare services were delayed or suspended, disproportionately impacting children with rare diseases such as leukodystrophies. Telemedicine facilitated continuity of care by reducing travel and associated costs, particularly for families in underserved areas [12]. Challenges in treatment adherence for patients with IEM are prevalent across all ages and groups and often intensify during adolescence and adulthood. This issue is particularly critical in conditions such as urea cycle disorders (UCDs) and maple syrup urine disease (MSUD), where poor dietary adherence can lead to acute metabolic crises, while in cases like phenylketonuria (PKU), insufficient dietary control can increase the risk of long-term complications [13]. For these families, telemedicine and digital tools, such as nutritional management apps (e.g., Metabolic Diet App Suite), are invaluable in facilitating adherence to personalized diets and treatment regimens [14]. The International Rare Diseases Research Consortium (IRDiRC) has outlined strategies to improve diagnosis and access to treatment through international collaboration, but obstacles remain, particularly for patients in rural areas or regions with limited healthcare resources [15,16]. In this context, telemedicine emerges as a crucial resource to address barriers to care. By providing remote support, it enables patient monitoring and fosters interdisciplinary collaboration among specialists, facilitating timely interventions even for those residing far from specialized centers [17]. Digital technologies not only reduce waiting times and physical distances but also improve access to diagnoses and treatments, enhancing continuous and personalized support for patients with rare diseases [18]. A study conducted at the Montreal Children’s Hospital on children with genetically determined leukodystrophies found that delays in in-person healthcare led to regressions in their physical abilities. Although these delays hindered symptom management, the introduction of telemedicine provided some relief by reducing travel needs and associated costs, crucial for families in rural or underserved areas. This example highlights the complexity of telemedicine for families with rare diseases, confirming the importance of a flexible, accessible care model, particularly during public health emergencies [12]. This review aims to analyze the role of telemedicine in improving access to care for patients with rare diseases. Compared to traditional care, telemedicine offers faster diagnosis, reduced waiting times, and more consistent, personalized support for this vulnerable population. It will also discuss the challenges to implementation, including technological, economic, and regulatory hurdles.

## 2. Materials and Methods

This narrative review was conducted to analyze the role of telemedicine in managing pediatric patients with rare diseases, with a specific focus on congenital neurological diseases, neuromuscular diseases, and inherited metabolic diseases. The analysis focused on improving access to care, therapeutic outcomes, and the potential of personalized nutritional interventions. A literature search was conducted to identify relevant articles focused on telehealth interventions in the nutritional management of children with rare disease aged 0 to 18 years. The authors independently conducted extensive literature research on PubMed (Medline) and Scopus databases, including articles published in the last 20 years. Only articles in English were included, focusing on pediatric patients diagnosed with rare neurological diseases or inherited metabolic disorders, and using telemedicine in addition to in-person visits at their reference center. Studies involving other pathologies, comorbidities, or patients older than 18 years were excluded. Keywords used in the search strategy are listed in Supplementary Table S1. Only articles that examined the use of telehealth interventions in patients with congenital neurological diseases, neuromuscular diseases, and inherited metabolic diseases for their management were included. Starting from a total of 1185 papers, of which 19 duplicates were removed, 1100 articles were excluded after title and abstract screening, as they did not focus on rare diseases of patients with severe neurological impairment or on the use of telemedicine in their management. The authors then reviewed the full texts of the remaining papers and finally selected 66 relevant articles, which were analyzed and included in the final review to provide a critical discussion. The flowchart diagram of paper inclusion is presented in Supplementary Figure S1. A narrative synthesis approach was used to summarize the results of the included studies. The results were organized according to the main outcomes, including the advantages and limitations of telemedicine in the management of rare diseases compared to traditional care, including the following: (A) Telemedicine Approaches in Rare Diseases; (B) Involvement of Multidisciplinary Teams; (C) Challenges and Limitations of Telemedicine in Rare Diseases; (D) Innovations and the Future of Telemedicine in Rare Diseases.

## 3. Results

### 3.1. Advantages and Limitations of Telemedicine in Management of Rare Diseases Compared to Traditional Care

Telemedicine offers several advantages over traditional care, particularly for patients with rare diseases. A key benefit is the increased accessibility to specialized consultations, crucial for managing rare conditions that require specific expertise. Remote consultations can reduce the burden of travel and associated costs, facilitating timely care access for families. For example, patients with conditions such as SMA have reported positive experiences with telemedicine, indicating comfort and perceived effectiveness in remotely managing their care [19]. This accessibility can be particularly beneficial for families residing in remote or underserved areas, where specialists may not be readily available. Studies have shown that telemedicine can effectively support the management of metabolic diseases, improving metabolic outcomes during challenging periods such as the COVID-19 pandemic [20]. Telemedicine can facilitate access to multidisciplinary teams, enabling patients to receive long-term care from various specialists without the burden of extensive travel. This approach enables a more individualized and adaptive management of rare diseases, responding to the evolving needs of each patient. Telemedicine has also proven useful for administering standardized scales aimed at assessing the functional impact of neurodegenerative diseases, such as leukodystrophies, bypassing the need for transporting patients with motor disabilities [21].

Another advantage of telemedicine is the ability to facilitate continuous monitoring and real-time adjustments to care plans. For diseases like phenylketonuria, where dietary adherence is critical, telehealth platforms can enable regular consultations with dieticians and physicians, promoting a more proactive approach to disease management.

Additionally, the integration of artificial intelligence (AI) in telemedicine represents a promising avenue for improving diagnostics for rare diseases, enabling faster and more accurate identification of conditions [22]. By leveraging machine learning algorithms and big data, AI can assist healthcare providers in recognizing clinical patterns and making informed decisions, thereby improving patient outcomes.

Despite these advantages, telemedicine has limitations that must be acknowledged. Firstly, technical issues, such as inadequate internet access or lack of familiarity with technology, can hinder effective communication and care delivery. Moreover, the inability to conduct physical examinations may lead to oversights of important clinical signs, which are critical in managing complex rare diseases. While telemedicine has proven to be a valuable tool during the COVID-19 pandemic, it also highlighted the limitations of virtual care, such as inadequate internet access, lack of technological proficiency, and difficulty in conducting physical exams. Despite progress in addressing rare diseases, significant challenges remain, including the need for better access to care [23].

In-person evaluations allow for comprehensive assessments that can sometimes identify signs which are not easily discernible through virtual consultations. Additionally, while telemedicine can enhance access to specialists, its full potential lies in being part of a well-coordinated healthcare strategy. Patients with rare diseases often require coordination between multiple healthcare providers, and telemedicine could complicate this process if not integrated into a comprehensive care strategy. Graessner et al. (2024) provided recommendations for optimal interdisciplinary management and healthcare settings for patients with rare neurological diseases, emphasizing the importance of collaboration among various healthcare professionals [24].

In conclusion, while telemedicine offers significant opportunities to enhance the management of rare diseases, it is essential to address its limitations to optimize patient care. An integrated approach, combining telemedicine with in-person visits, can help address complex health challenges associated with rare diseases, ensuring the continuity and comprehensiveness of care. Policymakers and healthcare systems must consider investment in telemedicine infrastructure to ensure equitable access, especially for underserved populations [25]. Therefore, while telemedicine can enhance care delivery, it must be integrated thoughtfully into existing healthcare structures to ensure that the unique needs of patients with rare diseases are met.

### 3.2. Telemedicine Approaches in Rare Diseases

#### Remote Consultations and Real-Time Monitoring: Use of Platforms and Devices in Rare Diseases

Platforms for remote consultations, combined with devices and apps for the real-time monitoring of clinical parameters, are reshaping healthcare delivery for rare diseases. The use of telemedicine is emerging as a critical resource in the management of rare diseases, which are characterized by low prevalence, complex medical needs, and often a fragmented geographical distribution of patients. Remote consultations and telehealth platforms help overcome logistical barriers, reduce waiting times, and improve access to highly specialized care that would otherwise be difficult to obtain (Table 1). According to the WHO’s recommendations, telemedicine is recognized as a primary strategy for providing, maintaining, and enhancing healthcare services disrupted by the COVID-19 pandemic [26]. The report by Omboni et al. highlights that the primary goal of telemedicine (49.7%) is to ensure integrated patient care by combining various services to provide diagnosis, treatment, monitoring, and rehabilitation [2]. The remote monitoring of patients in their home environments enables healthcare professionals to gain a more nuanced understanding of the social and environmental factors impacting their health. A critical factor in the advancement of telemedicine is the increasing availability of technology. According to the Pew Research Center’s 2021 report, 93% of Americans access the internet [3]. Additionally, 81% of Americans own smartphones, nearly 75% have desktop computers or laptops, and about 50% possess tablets or e-readers [27]. Today, telemedicine goes beyond simple phone consultations, leveraging innovations like smartphones, wearable devices, and biosensors to facilitate remote diagnostics and enhance care delivery. These tools include digital innovations such as smartphone cameras and video recorders, and specialized medical instruments like digital stethoscopes, ophthalmoscopes, otoscopes, and various biosensors. Remote patient monitoring (RPM), which involves collecting and transmitting patient-generated health data (PGHD) to healthcare professionals, has gained traction [28]. This approach supports proactive healthcare delivery, helping clinicians detect changes in patients’ health complications. Telemedicine is also extensively utilized within emergency medical systems. For instance, the transmission of ECGs is now a standard practice for assessing eligibility for invasive procedures, available in most ambulances [6]. Methods for remote patient registration focus on gathering health data through wearable sensors, mobile applications, or diagnostic devices like stethoscopes and ultrasound devices. These wearable devices capture information such as heart rate, oxygen saturation, respiratory rate, ECG readings, and body temperature [5]. Modern sensors even allow the remote evaluation of lung sounds and imaging, expanding diagnostic capabilities. Wearable body sensors networks represent a promising solution for remote healthcare systems, as noted by Qureshi et al. These devices collect comprehensive health-related data, which are stored on local servers for clinicians to access during decision-making processes [28,29]. However, the expertise available at specific healthcare provider (HCP) centers may not always suffice, driving the need for cross border e-health solutions [11]. The European Reference Networks (ERNs) address this need by enabling healthcare providers to share specialized knowledge [30]. A cornerstone of ERNs is the Clinical Patient Management System (CPMS), a secure digital platform for cross-border case discussions among clinicians [13]. CPMS facilitates the sharing of medical images, reports, and consultations via video meetings, enabling multidisciplinary discussions for rare and complex diseases [31]. The case discussions are conducted by opening “panels”, where the clinician seeking medical advice provides a comprehensive description of the clinical case. Sharing data, such as radiological images, test reports, and lab results, is possible through these panels, allowing specialists to easily offer their input during virtual consultations via both chat and video meetings. A final report summarizing all steps of the discussion and concluding remarks is generated, and the patient’s data can be stored in databases and registries, provided consent is given. In this context, CPMS goes beyond simply resolving cases: its capacity to store vast amounts of information turns it into a valuable digital database, useful for both research and data analysis. By maintaining patient data and enhancing the evidence-based understanding of rare diseases, it supports the sharing of information on ultra-rare conditions and aids in precise diagnosis. Since any functional study (clinical, phenotypic, imaging, biochemical, genetic, etc.) can be logged in the system, it serves as a source of functional data, offering unique scientific insights. As a result, this growing database could be used to help interpret data, including those linked to Variants of Uncertain Significance (VUS) [32]. One of the most notable advantages of the CPMS is its ability to enable healthcare providers across all ERNs to collaborate and share cases, thereby creating a multidisciplinary clinical and diagnostic environment that maximizes the likelihood of an accurate diagnosis. Despite its potential to improve the quality of healthcare delivery, the CPMS is often underutilized. Like other telemedicine tools, its steep learning curve and inherent system complexity hinder integration into daily practice. These issues have become even more evident in the COVID-19 era [33], where telemedicine has become an essential part of medical practice [34]. Suboptimal use of the CPMS may be due in part to insufficient understanding of how to navigate the system, coupled with the challenge of the platform being in English, which is necessary to facilitate cross-border communication across Europe [35]. Telemedicine is increasingly used as an everyday approach to facilitate the diagnosis and treatment of a heterogeneous set of clinical conditions, ranging from mild to critical cases [36]. Telemedicine solutions integrated with artificial intelligence enable the development of algorithms that assist in decision-making or guide patients and doctors through the diagnostic process. These algorithms can be used to evaluate overall health status and patient cohorts [37]. The involvement of multidisciplinary teams (e.g., doctors, nurses, psychologists, nutritionists) plays a pivotal role in leveraging telemedicine to enhance patient outcomes.

### 3.3. Involvement of Multidisciplinary Teams (Doctors, Nurses, Psychologist, Nutritionists)

**Figure 1:**
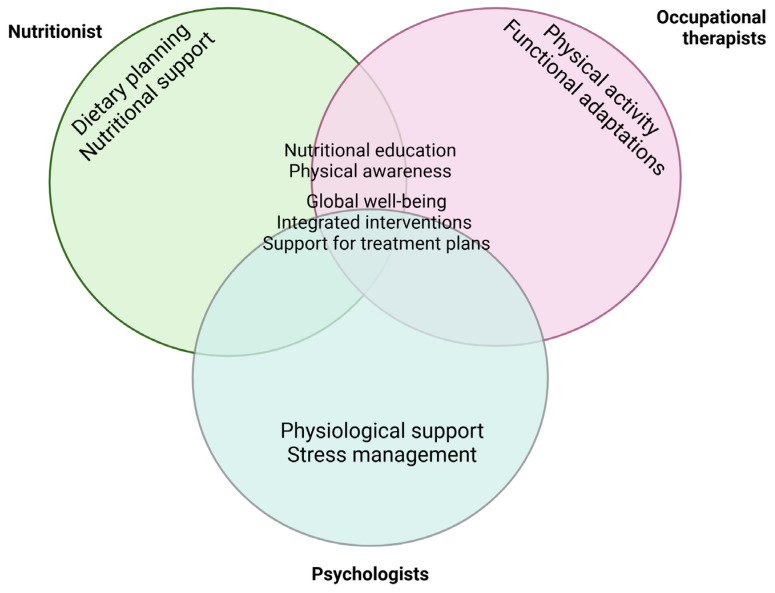
Overlapping areas of expertise and collaboration among nutritionists, occupational therapists, and psychologists in supporting children with neurological impairments, highlighting importance of multidisciplinary approach.

Nutritional assessment and interventions for children with neurological impairments (NIs) present significant challenges for healthcare providers. These interventions are critical components of comprehensive care and rehabilitation, aimed not only at promoting weight and linear growth but also at enhancing physiological and functional capacities. Nutritional issues in this population can stem from diverse causes, necessitating various interventions such as positioning, rehabilitation, dietary modifications, and pharmacological treatments. A multidisciplinary approach (Figure 1) is crucial in managing nutrition for children with NIs, involving collaboration among occupational therapists, psychologists, speech therapists, dietitians, physicians, and nurses. This integrated team approach facilitates personalized care tailored to each child’s evolving needs. Importantly, telemedicine serves as a vital tool in facilitating this collaboration, enabling real-time consultations and coordinated care [38]. Telemedicine not only enhances accessibility to care but also enables rapid adjustments to treatment plans, ensuring continuity even in emergencies or evolving clinical conditions. Through regular teleconsultations, patients and their families can receive continuous nutritional and psychological support, reducing the stress associated with hospital visits and improving their overall quality of life. Speech and language therapy often form the mainstay of treatment for children with neurodisabilities. Oral sensorimotor therapies aim to improve the individual and combined functioning of the lips, cheeks, tongue, and pharyngeal structures. However, studies indicate that while these therapies can improve feeding time, they may not significantly affect liquid swallowing abilities. Hirata and Santos [39] performed a systematic review on the rehabilitation of oropharyngeal dysphagia (OPD) in children with NIs and found limited evidence supporting therapeutic interventions [40]. The study [39] highlighted the severity of swallowing disorders, which can affect the preparatory, oral, pharyngeal, and oesophageal phases of swallowing. It identified rehabilitation methods such as the Bobath concept, the Castillo Morales concept, oral sensorimotor therapy, and continuing education. However, only a small percentage of articles (7.09%) addressed these issues, suggesting that, despite the increasing number of cases of cerebral palsy, research and therapeutic interventions for oropharyngeal dysphagia remain limited. The most common methods were oral sensorimotor therapy and continuing education, while the use of the Bobath and Castillo Morales concepts was less frequent. This highlights the need for a greater focus on rehabilitation for swallowing disorders in this population. Oral pharyngeal dysfunction is a significant factor contributing to reduced oral food intake, posing a risk for undernutrition. Many children with NIs experience trunk hypotonia, leading to poor posture that exacerbates swallowing difficulties. Surgical procedures for scoliosis, though necessary, may result in gastric dysmotility, potentially linked to sympathetic overstimulation [41]. Such complications underline the importance of integrating gastrointestinal and nutritional assessments into care plans. The involvement of a multidisciplinary team is essential in managing feeding for all children with NIs. While oral feeding should always be prioritized, safety remains paramount. In cases of severe oropharyngeal dysphagia, partial or full enteral feeding may be required, with close monitoring to adjust interventions as needed. Diet composition and texture modifications must be discussed with a specialized feeding therapist or dietitian [41,42] to ensure safe and efficient food intake. Beyond physical health, the psychological well-being of patients and families plays a crucial role in holistic care [35]. Nutritional challenges often coincide with emotional strain, significantly impacting family dynamics [43,44]. In conclusion, the presence of a multidisciplinary team ensures comprehensive and personalized care for children with NIs. The integration of telemedicine further optimizes this collaboration, breaking down geographical barriers and enabling real-time, patient-centered interventions. This approach improves nutritional outcomes, promotes emotional well-being, and enhances the overall quality of life for patients and their families.

### 3.4. Challenges and Limitations of Telemedicine in Rare Diseases

Despite the growing use of telemedicine and its potential to improve the management of rare diseases (RDs), several challenges persist that limit its full potential and hinder its widespread adoption. These include technical issues, difficulties in maintaining continuity of care, concerns about data security and privacy, economic and organizational barriers, and geographical and socioeconomic disparities in access to services [45,46,47].

#### 3.4.1. Technical Issues

Technical limitations are one of the main barriers to successful telemedicine implementation, particularly in rural areas where technological infrastructure, such as broadband networks and stable connections, is often inadequate. These deficiencies compromise the quality of interactions and limit access to reliable healthcare services [48].

Socioeconomic factors, such as education level, household income, and familiarity with digital tools, also significantly impact the adoption of telemedicine. Enhancing digital literacy, especially in underserved regions, is crucial in reducing anxiety and perceived barriers associated with these technologies, which would foster greater participation and improve the overall experience [49].

#### 3.4.2. Difficulty in Maintaining Continuity of Care

Ensuring continuity of care remains a critical challenge in the implementation of telemedicine, especially for RD patients requiring ongoing monitoring and multidisciplinary interventions. During the COVID-19 pandemic, many families experienced disruptions in traditional healthcare services, leading to diagnostic delays and worsening clinical conditions. While telemedicine provided valuable remote support, its limitations—such as the inability to perform comprehensive physical examinations and challenges in managing emergencies—highlighted the difficulties of delivering holistic care exclusively through digital means [12].

Telemedicine has also proven to be a useful tool for addressing access challenges to specialized care, ensuring continuity even in underserved settings. For example, in pediatric neurodevelopmental disorders, remote sessions have enabled sustained therapeutic support, improved caregiver skills, and ensured consistent access to specialist expertise [50]. Research demonstrates that telemedicine interventions can rival the effectiveness of in-person care, delivering significant improvements in patient outcomes such as alleviating health and behavioral challenges among children and expanding care accessibility in resource-limited settings [51].

#### 3.4.3. Concerns About Data Security and Privacy

Healthcare apps and mHealth tools, commonly used in telemedicine, raise significant concerns about data security and privacy. The extensive amount of sensitive information collected, transmitted, and analyzed highlights the need for clear, uniform regulations to safeguard patient confidentiality. For example, in the United States, the Health Insurance Portability and Accountability Act (HIPAA) ensures the protection of health information transmitted between covered entities, such as healthcare providers and insurance plans. However, information transmitted through apps or mobile devices not covered by HIPAA lacks equivalent protection, creating potential vulnerabilities [52].

In Europe, the General Data Protection Regulation (GDPR) establishes high privacy standards, but its variable application across countries leads to uncertainties in adopting mHealth tools [53]. Moreover, the regulation of health apps varies depending on their classification: some are strictly regulated, while others are not considered medical devices, leading to variability in user protections [45,52].

#### 3.4.4. Economic and Organizational Barriers

The cost-effectiveness of telemedicine remains a subject of debate. While evidence suggests that this technology can reduce some treatment costs, existing analyses often suffer from methodological shortcomings, such as limited sample sizes, the absence of randomized clinical trials, and insufficient standardized metrics. Additionally, broader social and organizational costs are often overlooked: most studies focus on healthcare system expenses while neglecting the economic impact on patients and their social networks [54].

Broader economic evaluations are crucial to accurately compare the benefits of telemedicine with those of traditional healthcare services. Further research should aim to identify strategies for improving efficiency and sustainability [55].

#### 3.4.5. Disparities in Access to Telemedicine in Different Geographical Areas

Geographical disparities pose a significant challenge to telemedicine implementation, particularly in rural or economically disadvantaged regions. In these areas, inadequate technological infrastructure, such as broadband access or suitable devices, often limits the ability to provide high-quality telemedicine services. Stable and reliable connectivity between patients and providers is critical for telemedicine’s success; however, meeting this requirement remains difficult in remote areas, negatively impacting service effectiveness [48].

The COVID-19 pandemic further highlighted these disparities. A study on Medicare beneficiaries in the United States found that 26.3% lacked digital access at home, making video telemedicine visits impossible. This issue was more pronounced among older adults, low-income families, and communities of color. Although initiatives like the Lifeline program have attempted to bridge these gaps by providing low-cost internet access, such programs often fail to address device affordability or provide training in technology usage, leaving many patients unable to utilize telemedicine services effectively [56].

### 3.5. Innovations and Future of Telemedicine in Rare Diseases

#### 3.5.1. Artificial Intelligence (AI) Including Machine Learning for Large Dataset Analysis

Emerging medical technologies are transforming healthcare through innovative interactions between doctors, patients, and medical devices, bringing numerous advantages to all stakeholders. Telemedicine’s greatest strength lies in its ability to overcome the barriers of distance and time, reaching a broader population of patients suffering from rare chronic diseases [48].

Today, smart healthcare systems rely on various technologies: sensors for clinical data acquisition, communication modules for data transmission, real-time databases for data storage, and applications for data processing and visualization [56,57]. Among these, artificial intelligence (AI) plays a crucial role, with machine learning (ML) as one of its key components for processing large datasets and extracting meaningful patterns.

Some examples of medical devices include the following:

- E-diaries: Apps or websites designed to track patients’ daily activities, symptoms, or physiological status in a digital format. Examples include tools for dietary tracking, growth and weight monitoring, and medication adherence.
- Remote patient monitoring (RPM): Wearable devices, mobile apps, or websites used to collect real-time patient data outside traditional healthcare settings and transmit it to a remote location. Examples include heart rates monitors, blood glucose monitors, drug level trackers, and breath ketone analyzers [6,58].

However, these approaches face several limitations, including the size and weight of the system or its components (especially to pediatric patients), battery life, usability across various settings, and the costs of data transmission over internet networks. Moreover, studies evaluating their effectiveness are limited, with inconsistent measures of device or app quality. Consumer devices and apps also lack universal regulation [57].

#### 3.5.2. Development of New Telemonitoring Tools and Digital Medical Devices

Current trends in telemonitoring and digital medical devices development focus on the following:

- Advancing small-scale sensors (e.g., nanoscale sensors) and improving communication technologies for efficient data transmission.
- Creating innovative cloud platforms for secure and efficient data extraction and decision-making.
- Enhancing data management systems to ensure robust privacy and security [59,60].

These innovations could enable the creation of large anonymized, open-source datasets for objectively evaluating the clinical presentation of rare diseases. This standardization of data could facilitate future studies while meeting regulatory requirements and gaining medical/ethical authorization [60,61].

#### 3.5.3. Future Perspectives and Potential Developments in Telemedicine and Remote Care

The diffusion of telemonitoring has led to the collection of vast amounts of data, necessitating advanced interpretation methods to make this information actionable [57]. One of the most significant future objectives is to optimize the application of AI and related advanced cognitive systems, such as ML, and processing the collected Big Data [59,62,63].

The term Big Data refers to large volumes of structured and unstructured information that traditional database systems cannot process effectively. Computational analyses of these datasets can uncover patterns, trends, and associations, enabling better data interpretation [57,63].

AI has the potential to assist clinicians and researchers in several ways.

- AI provides advanced analytical capabilities, allowing it to address questions related to diagnosis, prediction, and prescription that go beyond traditional methods. AI can outperform human experts in certain medical decision-making processes, achieving lower error rates. Additionally, AI is being applied to acquire processes and analyze medical images using innovative algorithms in different medical fields, for example, in cardiology [64].
- ML excels at recognizing complex patterns in different datasets (e.g., numerical, visual, and textual) and processing large datasets at unparalleled speeds and accuracy. This capability can enhance diagnostic criteria by identifying key features across varied patient populations [62]. The process typically involves structuring raw data into an initial database, followed by analysis to identify and apply significant variables to ML algorithms. These methods have applications in neurology, immunology, allergology, and cardiology [64,65,66].
- In silico models are a burgeoning field that uses mechanistic models, as opposed to purely statistical methods, to stimulate phenomena of interest. A key advantage is the generation of virtual populations for in silico clinical trials (ISCTs). These trials refine inclusion and exclusion criteria, allow testing on underrepresented groups (e.g., pediatrics), and serve as virtual control arms, offering valuable insights for real-world clinical trials [63].

Health technologies are set to become an integrated part of future healthcare systems, delivering personalized care and preventive measures for patients with rare chronic diseases.

While the integration of advanced technologies appears inevitable, it is crucial for healthcare to remain cautious. Artificial intelligence systems are not infallible, and recognizing their limitations for intelligent integration into medical practice is essential [59,61,63].

## 4. Discussion

Overall, this narrative review based on a systematic search of the literature underscores the transformative role of telemedicine in the management of rare diseases, particularly in pediatric patients with complex neurological and metabolic conditions. By bridging geographical and temporal gaps, telemedicine enhances access to specialized care, facilitates multidisciplinary collaboration, and supports personalized treatment approaches. The integration of wearable devices, advanced sensors, and cloud-based platforms has further expanded the potential of telemedicine, enabling real-time monitoring and early intervention. Additionally, AI-driven tools are proving useful in analyzing complex clinical datasets, optimizing decision-making processes, and refining predictive models.

Despite these advancements, significant challenges remain. The lack of standardized regulations for digital health solutions, disparities in technological infrastructure, and concerns regarding data security and privacy must be carefully addressed. Furthermore, while AI and machine learning hold great promise, their widespread clinical application requires rigorous validation to ensure accuracy, reliability, and ethical compliance.

**Figure 2:**
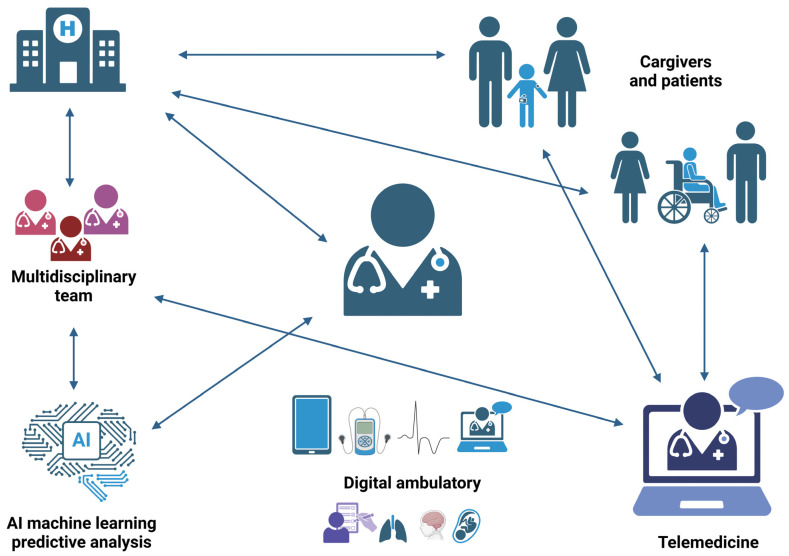
Management of pediatric patients with rare diseases: telemedicine, multidisciplinary teams, and artificial intelligence to enhance communication and clinical outcomes.

Looking ahead, the future of telemedicine in rare disease management appears highly promising. Emerging technologies, including in silico models for clinical trials, could revolutionize diagnostic and therapeutic strategies, particularly for underserved populations such as pediatric patients (Figure 2). Establishing open-access datasets and improving data interoperability will be critical in driving further research and innovation. Ultimately, while challenges persist, a responsible and evidence-based adoption of these technologies has the potential to significantly enhance patient care, optimize clinical outcomes, and improve the quality of life for individuals affected by rare diseases.

## 5. Conclusions

In conclusion, the integration of telemedicine with emerging technologies offers a unique opportunity to improve care for patients with rare diseases. By addressing existing limitations and fostering collaboration among stakeholders, telemedicine can progress toward a future in which personalized, accessible, and effective care becomes the standard. This evolution will not only improve health outcomes but also the quality of life of patients and their families.

## Figures and Tables

**Table 1:** Comparison between traditional approaches and telemedicine in management of rare diseases, highlighting advantages in terms of accessibility, monitoring, and multidisciplinary collaboration.

Aspect	Telemedicine	Traditional Treatment
Accessibility	High (remote access)	Limited (in person visits)
Timeliness	Quick (immediate consultations)	Long (waiting for appointments)
Costs	Lower (less travel expenses)	Higher (travel costs)
Monitoring	Continuous (real-time feedback)	Periodic

## Supplementary Materials

The following supporting information can be downloaded at: https://www.mdpi.com/article/10.3390/nu17030455/s1. Figure S1: Flowchart process of articles selection; Table S1: Keywords used in search strategy.
