# Supplementary material for: Telemedicine for Personalized Nutritional Intervention of Rare Diseases: A Narrative Review on Approaches, Impact, and Future Perspectives
Source: Nutrients. 2025 Jan 26;17(3):455. doi: 10.3390/nu17030455 (PMC11820740; doi:10.3390/nu17030455)
Supplement: Supplementary file 1 [file nutrients-17-00455-s001.zip › Outcomes.pdf]

**Outcomes**	**Keywords**
Implementation of healthcare with telemedicine in rare diseases	“telemedicine” OR “telehealth” OR “telemonitoring” AND “rare disease” AND “neurological disorder” OR “neurological impairment” AND “inherited metabolic disease” OR “ereditary metabolic disease”
Advantages and limitations of telemedicine compared to traditional care	“Telemedicine” AND “traditional care” OR “in-person care” AND “advantages” AND “limitations” AND “rare diseases”
Multidisciplinary approach to rare diseases	“Multidisciplinary approach” OR “multidisciplinary team” AND “rare disease” AND “medical doctor” AND “nutritionist” OR “dietitian” AND “psychologist” AND “physiotherapist”
Innovations and the Future of Telemedicine in Rare Diseases	“Artificial intelligence” OR “machine learning” OR “new technologies” AND “rare disease” AND “neurological impairment” AND “inherited metabolic disease”

Supplementary Table S1: Keywords used in the search strategy.
