# Supplementary figures and images for: Telemedicine for Personalized Nutritional Intervention of Rare Diseases: A Narrative Review on Approaches, Impact, and Future Perspectives
Source: Nutrients. 2025 Jan 26;17(3):455. doi: 10.3390/nu17030455 (PMC11820740; doi:10.3390/nu17030455)

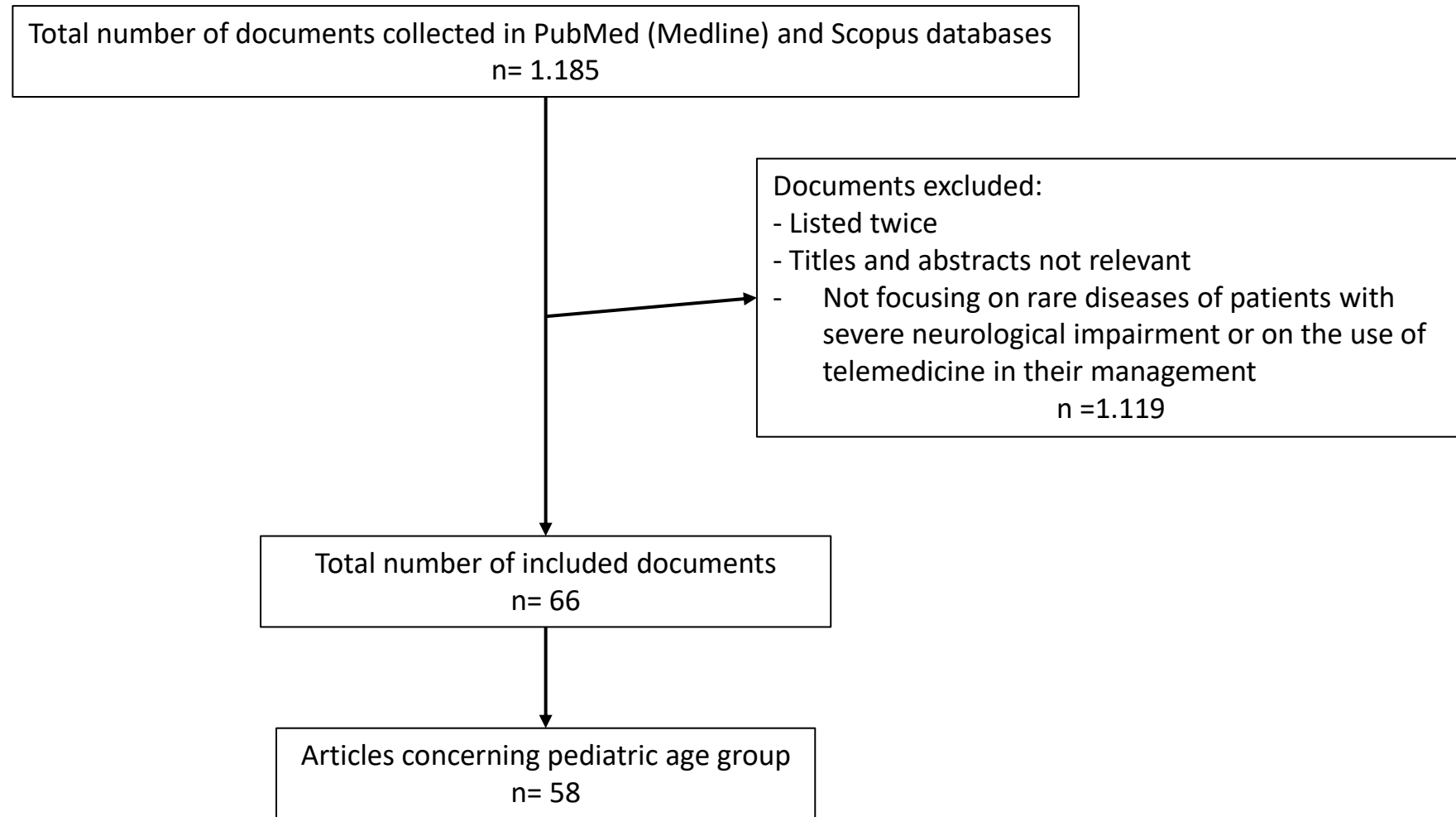

Supplementary Figure S1: flowchart diagram of paper inclusion.

Supplement: Supplementary file 1 [file nutrients-17-00455-s001.zip › Flowchart.pdf]
